# Larvicidal Activities of *Allium sativum* L. and *Zingiber officinale* Rosc. Extracts against Filariasis Vectors in Hadiya Zone, Ethiopia

**DOI:** 10.1155/2023/6636837

**Published:** 2023-05-31

**Authors:** Anmut Assemie, Temam Gemeda

**Affiliations:** ^1^Department of Biology, Wachemo University, P.O. Box 667, Hossana, Ethiopia; ^2^Department of Biotechnology, Wachemo University, PO Box 667, Hossana, Ethiopia

## Abstract

Mosquitoes present an immense threat to millions of people worldwide and act as vectors for filariasis disease. The objective of the study was to determine the effect of *Allium sativum* and *Zingiber officinale* extracts against filariasis vectors. The larvae were collected from the breeding site by using standard procedures for identification and larvicidal activities. Twenty grams (20 g) from each (*Allium sativum* and *Zingiber officinale*) were extracted separately by aqueous, ethanol, and methanol solvents. The phytochemical analysis was determined in the crude sample by using standard methods. Then, larvicidal effects were determined by introducing 10 larvae of the vectors to the concentrations of 250 ppm, 500 ppm, and 750 ppm of the crude sample, and data were subjected to probit analysis to determine the LC_50_ and Chi-squared test to check the significance of the mortality by R software. *Anopheles funestus*, *Anopheles gambiae* s.l., *Anopheles pharoensis*, *Culex antennatus*, and *Culex quinquefasciatus* were the filariasis vectors identified during the study period. The presence of phytochemical tests such as anthraquinones, flavonoids, glycosides, phenol, saponin, steroids, tannin, and terpenes was obtained. The larvicidal effects of the selected plant extracts ranged from 0%-100%. The lowest LC_50_ (53 ppm) was observed for *A. sativum* methanol test extract against *Cx. quinquefasciatus*. Ethanol extracts of *A. sativum* have a significant effect on *An. funestus* (*X*^2^ = 7.5, *p* = 0.02352) and *Cx. quinquefasciatus* (*X*^2^ = 10.833, *p* = 0.0.0044), whereas aqueous extracts have a significant effect only on *An. gambiae* s.l. (*X*^2^ = 7.0807, *p* = 0.029. Ethanol extracts of *Z. officinale* have a significant effect only on the mortality of *An. pharoensis* (*X*^2^ = 7.0807, *p* = 0.029), but methanol and aqueous extracts have no significant effect against filariasis vectors. In conclusion, *A. sativum* have a high toxic effect than *Z. officinale* extract against filariasis vectors in all type of solvents. So using those plant extracts is the best to reduce the risk of the synthetic chemical on nontarget organisms and the environment, in addition to the control of mosquito-borne diseases, but further studies will be conducted to evaluate the toxicity at different stages of the vectors.

## 1. Introduction

Mosquitoes are the primary vectors of filariasis and play a key role in the spread of vector-borne diseases such as chikungunya, dengue fever, Japanese encephalitis, malaria, and yellow fever. Mosquito-borne diseases are a major source of illness and death worldwide, especially in tropical and subtropical countries [1].

Lymphatic filariasis is a tropical parasitic illness that affects the arms, legs, male genitals, and female breasts, causing permanent disability. Lymphatic filariasis is a disease that is prevalent in developing countries and causes a problem in sub-Saharan Africa, Asia, South and Central America, and Pacific Island countries [2]. About 1.3 billion people that live in 72 countries around the world were exposed to lymphatic filariasis disease [3, 4], the majority of whom are the poorest [5], and at least 120 million people live in 73 countries that include Africa, India, and Southeast Asia [6].

In Ethiopia, the first lymphatic filariasis was recorded in 1971 [7], making Ethiopia the fourth-highest burden country in sub-Saharan Africa [7]. Lymphatic filariasis was endemic in Gambella; Benishangul-Gumuz; Southern Nations, Nationalities, and People's Region; Amhara; and Oromia [8]. South Arsi, Benatsemay and Selemago, Teltele, Simada, and Tach-Gayint were the five lymphatic filariasis-endemic districts in Ethiopia [9].

The prevention and control of mosquito populations using chemical insecticides had some advantages based on their fast action and easy application at the beginning. But the continuous use of chemical insecticides for the reduction of the mosquito population led to negative effects on public health and agriculture such as the rapid development of resistant mosquito strains, harmful impacts on beneficial organisms, environmental pollution, and toxic hazards to mammals [10]. Alternatively, plant extracts have been used to manage mosquito populations due to their repellent or insecticidal properties, and the combination of different components or biocidal organisms has been evaluated to guarantee efficacy and safety in insect control [11, 12]. Microbe-based control agents provide an alternative to conventional pesticides and insecticides, as they can be more targeted than synthetic insecticides [10]. Phytochemicals with mosquitocidal potential are now recognized as potent alternative insecticides to replace synthetic insecticides in mosquito control programs because plants contain a wide range of potential larvicidal phytochemicals (anthraquinones, flavonoids, glycosides, phenol, saponin, steroids, tannin, and terpenes.) that are target specific, rapidly biodegradable, ecofriendly, and less toxic to human health [13, 14].

*Z. officinale* and *A. sativum* are of those plants that were seriously investigated over several years and used for centuries to fight infectious diseases and have antifungal, antiviral, antibacterial, anthelmintic, antiseptic, and anti-inflammatory properties [15–17]. Natural pesticides in the reduction of vector populations have received more attention [18] because there is a growing interest in producing pesticides derived from plants as an alternative to chemical insecticides. As far as we know, there are no previous studies about the potential use of *A. sativum* and *Z. officinale* extracts against identified filariasis vectors (*An. funestus*, *An. gambiae* s.l., *An. pharoensis*, *Cx. antennatus*, and *Cx. quinquefasciatus*) in the study area. Therefore, this study aims at evaluating the aqueous, ethanol, and methanol extracts of *A. sativum* and *Z. officinale* extracts against the larvae of *An. funestus*, *An. gambiae* s.l., *An. pharoensis*, *Cx. antennatus*, and *Cx. quinquefasciatus*.

## 2. Materials and Methods

### 2.1. Description of Study Area

The current study was conducted in the Hadiya Zone, the southern region of Ethiopia. Hadiya Zone is bordered on the south by Kembata Tembaro, on the southwest by the Dawro Zone, on the west by the Omo River which separates it from the Oromia Region and the Yem special woreda, on the north by Gurage, on the northeast by Silt'e, and the east by the Alaba special woreda; the woredas of Mirab Badawacho and Misraq Badawacho form an exclave separated from the rest of the zone by the Kembata Tembaro Zone. Hadiya Zone is located 232 km far from Addis Ababa and situated between 7° 39′ 59.99^″^ N latitude and 37° 44′ 59.99^″^ E longitude. Topographically, the zone lies within an elevation range of 1500 to 3000 meters above sea level. The zone has three agroecological zones: highland (23.7%), midaltitude (64.7), and lowland (11.6%). The annual average temperature of the zone is 22.02°C, and the mean annual rainfall is 1260 mm [19].

### 2.2. Collection of Filariasis Vector's Larvae

The study site was selected purposely based on altitude and temperature which are located between 1250 m and 2500 m above sea level and at a temperature between 21 and 29°C. The cross-sectional entomological collection was conducted in different breeding sites of filariasis vectors in the Shashago district, Hadiya Zone, Ethiopia. The larval collection was carried out to identify the species and to determine the presence of filariasis vectors in the study area, in addition to larvicidal tests. During larval collection, a standard dipper (350 ml) was used to make dips depending on the size of the larval breeding habitats. The dipper was lowered at 45° until one side was just below the water. Then, by using the water from the natural habitat, larvae were poured into plastic trays (75 × 75). The collected larvae were identified using morphological identification keys [20].

### 2.3. Collection and Preparation of Plant Extract

Fresh samples of *Z. officinale* and *A. sativum* were collected from Hossana main market, Hadiya Zone. The plants were authenticated by a botanist at the Department of Biology. The fresh *A. sativum* (garlic) cloves and *Z. officinale* (ginger) rhizomes were washed with tap water and peeled so that the main seed was exposed, and it is slashed into smaller sizes to ease the drying process. The slashed *Z. officinale* and *A. sativum* were taken to the biology laboratory for drying which was spread on a newspaper and placed in an open cabinet. The dried *Z. officinale* and *A. sativum* were pounded into powder form. Twenty grams (20 g) of the powder from each, *Z. officinale* and *A. sativum*, were soaked in 200 ml of distilled water, ethanol, and methanol separately.

The flasks were incubated at room temperature for 48 hours with shaking at 120 rpm. The crude extracts were centrifuged for 10 minutes at 25°C. The methanol and ethanol extracts were evaporated at 50°C, while the aqueous extracts were evaporated at 80°C in a rotary evaporator. All dried extract samples were dissolved in distilled water separately to the final concentration and centrifuged again to remove the undissolved residues. Each aqueous, ethanol, and methanol extract were named separately. The extract was then stored in labeled specimen bottles for larvicidal assay [16].

### 2.4. Phytochemical Analysis

The qualitative phytochemical analyses of the components that have a toxic effect on larvae were carried out using standard methods [21]. Methanol, ethanol, Mayer's reagent, glacial acetic acid, ferric chloride, concentrated sulphuric acid, concentrated hydrochloric acid, Molisch's reagent, ammonia, chloroform, distilled water, and iodine were the reagent required for the qualitative phytochemical screening of the crude extract [21].

### 2.5. Larvicidal Bioassay

The required crude extracts (25, 50, and 75 mg) of *Z. officinale* and *A. sativum* were set aside. For each treatment, three samples were needed to obtain three trials. Finally, 25, 50, and 75 mg of each sample were separately mixed with 100 ml of distilled water. For each sample, 100 ml of distilled water and malathion were used as a negative and positive control, respectively. Ten-fourth instar larvae of *Anopheles funestus*, *Anopheles gambiae* s.l., *Anopheles pharoensis*, *Culex antennatus*, and *Culex quinquefasciatus* were introduced in each glass beaker of 250 ml capacity containing different concentrations of test solutions. Larvae were considered dead if they showed no sign of movement even after being introduced into the glass that contains test solutions [22]. The treatments were observed after 6 h.

### 2.6. Statistical Analysis

After the bioassay study, all the observations become summarized and prepared for the inferential test, and the percentage of the mortality is calculated as follows:

(1) $$\begin{array}{c} \text{Percentage of mortality}=\frac{\text{number of dead larvae }}{\text{ number of larvae introduced}}\times 100. \end{array}$$

The average mortality data were subjected to probit analysis for calculating LC_50_ using R software. The mean values and standard deviations were calculated from replication data. Chi-squared values were calculated using the R version 4.2.1 (2022) (statistical software package) to find the regression equation values. *p* < 0.05 was considered to be statistically significant.

## 3. Results

### 3.1. Species of Filariasis Vectors

A total of 1012 filariasis vectors belonging to two genera *Anopheles* and *Culex* were collected from the study area during the study periods. From the collected filariasis vectors, the *Anopheles* become the highest in number (*n* = 621; 61.37%), while *Culex* was the lowest in number (*n* = 391; 38. 63%). The most dominant filariasis vectors which were identified as *Anopheles* belonged to *An. gambiae* s.l. (*n* = 221; 35.59%) followed by *An. pharoensis* (*n* = 207; 33.33%), while the lowest number belonged to *An. funestus* (*n* = 193; 31.08%). Among the *Culex* (*n* = 391), *Cx. antennatus* (*n* = 190; 48.59%) and *Cx. quinquefasciatus* (*n* = 201; 51.41%) were identified as filariasis vectors during the study period in the study area as indicated in Table 1. *An. gambiae* s.l. was the most predominant species identified as a filariasis vector in the study area during the study period.

### 3.2. Phytochemical Screening

The analysis of the aqueous, ethanol, and methanol extracts of *A. sativum* and *Z. officinale* showed positive results for eight and six phytochemical tests, respectively. The bioactive compounds present in the aqueous, ethanol, and methanol extracts of *A. sativum* were saponin, tannin, phenol, anthraquinones, flavonoid, glycosides, steroids, and terpenes, whereas tannin, flavonoid, glycosides, steroids, and terpenes were present in the *Z. officinale* aqueous, ethanol, and methanol extracts as indicated in Table 2.

### 3.3. Larvicidal Activities of *Allium sativum* L.

The effects of the *A. sativum* aqueous, ethanol, and methanol extracts were tested at 250 ppm, 500 ppm, and 750 ppm and showed mortality against the filariasis vector larvae after 6 h. In contrast to the crude extract of the selected plants, larva mortality was not observed in the negative control, while the positive control exhibited 100% larva mortality. The mortality observed in the aqueous, ethanol, and methanol extracts of *A. sativum* ranged from 0%-90% as shown in Table 3. The mortality of filariasis vectors due to the aqueous, ethanol, and methanol extracts of *A. sativum* increases as the concentration increases except for the methanol extract of *A. sativum* against *Cx. quinquefasciatus*. The highest mortality was observed in *An. gambiae* s.l., *An. funestus*, and *Cx. antennatus* due to methanolic extract and *Cx. quinquefasciatus* due to ethanol extract at a concentration of 750 ppm. The aqueous extract of *A. sativum* does not show any effect against *An. pharoensis* and *Cx. antennatus* at concentrations of 250 ppm and 500 ppm.

The tests of ethanol extract of *A. sativum* have no significant effect on the mortality of *An. gambiae* s.l. (*X*^2^ = 3.8095, *p* = 0.1489), *An. pharoensis* (*X*^2^ = 3.7321, *p* = 0.1547), and *Cx. antennatus* (*X*^2^ = 5.0893, *p* = 0.0785) but have a significant effect on *An. funestus* (*X*^2^ = 7.5, *p* = 0.02352) and *Cx. quinquefasciatus* (*X*^2^ = 10.833, *p* = 0.0.0044). The tests of aqueous extracts of *A. sativum* have a significant effect only on *An. gambiae* s.l. (*X*^2^ = 7.0807, *p* = 0.0.029), whereas methanol extracts of *A. sativum* have no significant effect on the mortality of *An. gambiae* s.l. (*X*^2^ = 3.8095, *p* = 0.1489), *An. pharoensis* (*X*^2^ = 3.333, *p* = 0.1889), *An. funestus* (*X*^2^ = 1.92, *p* = 0.3829), *Cx. antennatus* (*X*^2^ = 2.6087, *p* = 0.2713), and *Cx. quinquefasciatus* (*X*^2^ = 0.2678, *p* = 0.8747).

The ethanol extracts of *A. sativum* have LC_50_ values of 754 ppm, 697 ppm, 564 ppm, 422 ppm, and 384 ppm in *An. gambiae* s.l., *An. pharoensis*, *An. funestus*, *Cx. antennatus*, and *Cx. quinquefasciatus*; the aqueous extract of *A. sativum* has LC_50_ values of 736 ppm, 768 ppm, 726 ppm, 768 ppm, and 754 ppm in *An. gambiae* s.l., *An. pharoensis*, *An. funestus*, *Cx. antennatus*, and *Cx. quinquefasciatus*, and methanol extract has LC_50_ values of 262 ppm, 340 ppm, 131 ppm, 196 ppm, and 53 ppm in *An. gambiae* s.l., *An. pharoensis*, *An. funestus*, *Cx. antennatus*, and *Cx. quinquefasciatus*, respectively, as indicated in Table 4.

### 3.4. Larvicidal Activities of *Zingiber officinale* Rosc.

The aqueous, ethanol, and methanol extracts of *Z. officinale* showed a positive effect on filariasis vectors at the larvae stage. The lowest and the highest mortality were 0% and 90% at concentrations of 250 ppm and 750 ppm, respectively. As compared to the aqueous and ethanol extracts of *Z. officinale*, the methanol extract of *Z. officinale* was most effective against filariasis vectors. The highest mortality (90%) due to crude extracts of *Z. officinale* was observed in *An. funestus* and *Cx. antennatus*, whereas the lowest mortality was recorded in *An. gambiae* s.l. at a concentration of 250 ppm and 500 ppm, in *An. pharoensis* and *Cx. antennatus* at a concentration of 250 ppm.

The aqueous, ethanol, and methanol extracts of *Z. officinale* show a toxicity effect against the larva of *An. gambiae* s.l. (LC_50_ = 781 ppm, 924 ppm, 355 ppm), *An. pharoensis* (LC_50_ = 1940 ppm, 728 ppm, 393 ppm), *An. funestus* (LC_50_ = 4259 ppm, 415 ppm, 195 ppm), *Cx. antennatus* (LC_50_ = 919 ppm, 1080 ppm, 27 ppm), and *Cx. quinquefasciatus* (LC_50_ = 1679 ppm, 501 ppm, 251 ppm) as indicated in Table 4. In this study, the aqueous extract of *Z. officinale* does not show an effect against *An. gambiae* s.l. at concentrations of 250 and 500 ppm and *An. pharoensis* and *Cx. antennatus* at a concentration of 250 ppm.

The tests of aqueous extract of *Z. officinale* have no significant effect on the mortality of all tested filariasis vectors (*An. gambiae* s.l. (*X*^2^ = 2.069, *p* = 0.3554), *An. pharoensis* (*X*^2^ = 1.0714, *p* = 0.5853), *Cx. antennatus* (*X*^2^ = 4.0385, *p* = 0.1328), *An. funestus* (*X*^2^ = 0.48, *p* = 0.7866), and *Cx. quinquefasciatus* (*X*^2^ = 0.2870, *p* = 0.8663)). Methanol extract of *Z. officinale* have also no significant effect on the mortality of all filariasis vectors. But the tests of ethanol extract of *Z. officinale* have a significant effect only on the mortality of *An. pharoensis* (*X*^2^ = 7.0807, *p* = 0.029), but not in other filariasis vector larvae as indicated in Table 4.

## 4. Discussion

The identified lymphatic filariasis vectors in the present study were *An. gambiae* s.l., *An. pharoensis*, *An. funestus*, *Cx. antennatus*, and *Cx. quinquefasciatus*, which group under *Anopheles* and *Culex* genera in the study area. The occurrence of filariasis vectors in the study area was low as compared to the occurrence of filariasis vectors in other countries/study areas which were *Culex*, *Anopheles*, *Mansonia*, *and Aedes* [4, 23–26]. This variation may be due to the study period/season, way of larvae collection, time of larvae collection, way of identification, breeding sites, stage of identification, and biotic and abiotic factors.

The crude extract which is obtained from plant parts like leaves, roots, flowers, bark, seed, and fruits has been used as an alternative insecticide. Secondary metabolites derived from plant extract are used as larvicides, ovicides, adulticides, insect growth regulators, repellents, and oviposition attractants for the prevention and control of mosquito-borne disease transmission from individual to individual, community to community, and country to country because of their low cost, risk-free feature, and easy to manage [27, 28]. The crude extracts that have biological activities contain bioactive compounds which are chemical substances responsible for the mortality of half (50%) of the test organism [29, 30].

Different phytochemical compounds like saponin, tannin, phlobatannin, phenol, anthraquinones, glycosides, steroids, terpenes, and flavonoid were screened in the *A. sativum* and *Z. officinale* extracts. The aqueous, ethanol, and methanol extracts of *A. sativum* and *Z. officinale* show the presence/absence of those secondary bioactive compounds which have a positive response for the control and prevention of mosquito-borne diseases. Similar to the studies conducted, phytochemical screening of the crude extract of plants shows the presence/absence of anthraquinones, flavonoid, glycosides, phenol, phlobatannin, saponin, tannin, steroids, and terpenes, which have different biological activities [31–33].

The aqueous, ethanol, and methanol extracts of *A. sativum* against filariasis vector larvae show the least median lethal concentration and highest toxicity effect as compared to the extract of *Z. officinale* that was similar to the ethyl acetate extract of *Aspergillus tamarii* fungus against *Ae*. *aegypti* and *Cx. quinquefasciatus* mosquitoes, with the least LC_50_ and LC_90_ values [34].

In the present study, *A. sativum* and *Z. officinale* extracts do not show larvicidal effects before 6 hours but have an effect against larvae after 6 hours, which differs from other studies that showed larvicidal effect after 24 hours [35–39]. Negative control treatments do not show mortality, but positive control treatments showed 100% mortality of the filariasis larvae. The mortality of filariasis vectors due to the aqueous, ethanol, and methanol extracts of *A. sativum* ranged from 0%-90% at concentrations of 250 ppm, 500 ppm, and 750 ppm which was the most effective as compared to others [39]. The mortality of filariasis larvae by the aqueous, ethanol, and methanol extracts of *A. sativum* and *Z. officinale* was recorded, and the result related to the biological activities of various plant extracts by the previous researchers [36, 40, 41].

The aqueous, ethanol, and methanol extracts of *A. sativum* and *Z. officinale* were effective against the larvae of *Cx. quinquefasciatus* similar to the leaf extract of *Typhonium trilobatum* [42]; leaf, flower, and whole plant extract of *Jatropha curcas*, *Hyptis suaveolens*, *Abutilon indicum*, and *Leucas aspera* [36]; the leaf extract of *Mesua ferrea* [43]; fruit extract of *Croton caudatus*; flower extract of *Tiliacora acuminate* [44]; flower extract of *Tagetes erecta* [45]; *Azadirachta indica* oil formulation [40, 46]; the leaf extract of *Millingtonia hortensis* [47]; leaf extract of *Tagetus minuta*, *Ageratum conyzoides*, and *Jatropha curcas* [48]; and leaf extract of *Cucurbitaceous* plant [38].

In the present study, the mortality of *An. gambiae* s.l. by the aqueous, ethanol, and methanol extracts of *A. sativum* and *Z. officinale* was 0-100%, which was related to the N-hexane and chloroform extracts of *Annona senegalensis* against *An. gambiae* and *Cx. quinquefasciatus* at 2500, 1250, 625, and 312.5 ppm concentrations [49], aqueous extract of *Moringa oleifera* against *An. gambiae* s.l. larvae at 2000 ppm concentration [50], and aqueous and ethanol leaf extracts of *Hyptis suaveolens* against egg and larvae of *An. gambiae* at 1000, 500, 100, 50, and 5 *μ*g/m concentrations [51], but *Azadirachta indica*, *Cyperus alternifolius*, *Lupinus luteus*, *Lactuca sativa*, *M. alternifolia*, and *Persea americana* show 95-100% mortalities against *Cx. pipiens* [52, 53].

The mortality of *Cx. quinquefasciatus* was ranged from 10%-100%, which was in accordance to the larvicidal effects of hexane, chloroform, ethyl acetate, and methanol extracts of the leaf, flower, and whole plant of *Jatropha curcas*, *Hyptis suaveolens*, *Abutilon indicum*, and *Leucas aspera* at 100–500 ppm concentration [36]; hexane, ethyl acetate, and methanol dried leaf and bark extracts of *Ocimum gratissimum*, *Gleditsia triacanthos*, *Eucalyptus globulus*, and *Azadirachta indica* at 4.69 to 1000 mg/l concentration [54]; petroleum ether, chloroform, ethyl acetate, and methanol leaf extracts of *Melia dubia* and *Swietenia mahagoni* at concentrations of 62.5, 125, 250, 500, and 1000 mg/l [41]; petroleum ether and N-butanol extracts of dried whole plant of *Cassia occidentalis* at concentrations of 200 and 300 ppm [55]; and methanol and ethyl acetate leaf, seed, and stem extracts of *Acacia concinna*, *Cassia siamea*, *Coriandrum sativum*, *Cuminum cyminum*, *Lantana camara*, *Nelumbo nucifera*, *Phyllanthus amarus*, *Piper nigrum*, and *Trachyspermum ammi* against *An. stephensi* and *Cx. quinquefasciatus* at 62.5, 125, 250, and 500 ppm concentrations [56].

*A. indica* ethanolic extract is a good insecticide against *An. stephensi* larvae [57, 58]. *A. indica* oil formulation containing 0.15% azadirachtin was tested in three mosquito species, *Ae. aegypti*, *An. stephensi*, and *Cx. quinquefasciatus*, and the median lethal concentrations were 1.7, 1.6, and 1.8 ppm, respectively [35]. The acetone extracts of *Avicennia marina* have high mortality against *Cx. quinquefasciatus* (LC_50_ = 0.197 mg/ml; LC_90_ = 1.5011 mg/ml), *Anopheles stephensi* (LC_50_ = 0.176 mg/ml; LC_90_ = 3.6290 mg/ml), and *Aedes aegypti* (LC_50_ = 0.164 mg/ml; LC_90_ = 4.3554 mg/ml) [59]. In another study, 32% *A. indica* seed oil (an equivalent of 0.03% azadirachtin), after 8 days of exposure, showed a median lethal concentration (LC_50_) of 10.7 ppm in *An. gambiae* larvae. In the present study, the *A. sativum* and *Z. officinale* extracts have a less toxic effect against filariasis vector larvae, because the median lethal concentration (LC_50_) value is higher than the lethal concentrations previously determined on *An. gambiae* and *Cx. quinquefasciatus* larvae.

The current results showed that the most toxic crude extract with minimum median lethal concentration values against *Cx. quinquefasciatus* and *An. funestus* was methanol extracts of *A. sativum* and *Z. officinale*, respectively. *Z. officinale* oil had ovicidal efficacy against *Anopheles stephensi*, *Ae. aegypti*, and *Cx. quinquefasciatus*. The oil exhibited larvicidal activity against *Cx. quinquefasciatus*, *Cx. tritaeniorhynchus*, and *An. subpictus* with LC_50_ 50.78, 98.83, and 57.98 ppm, respectively [60].

## 5. Conclusion

The aqueous, ethanol, and methanol extracts of *A. sativum* and *Z. officinale* were effective in the control of filariasis vectors in the larvae stage at field conditions due to the presence of anthraquinones, flavonoids, glycosides, phenol, saponin, steroids, tannin, and terpenes, which have biological activities in the test organisms. Of the three types of extracts, methanol extract of *A. sativum* is more effective against filariasis vectors with low lethal concentration. In general, the aqueous, ethanol, and methanol extracts of *A. sativum* and *Z. officinale* reduce the population of *An. funestus*, *An. gambiae* s.l., *An. pharoensis*, *Cx. antennatus*, and *Cx. quinquefasciatus*; therefore, the use of their crude extract would decrease mosquito-borne diseases by reducing the population of mosquitoes.

## 6. Recommendation

The quantitative screening, purification, and determination of the structure of active ingredients of the aqueous, ethanol, and methanol extracts of *A. sativum* and *Z. officinale* are necessary for their wide use in mosquito-borne disease prevention and control. In general, further studies will be done on the general toxicant of aqueous, ethanol, and methanol extracts of *A. sativum* and *Z. officinale* against various stages of mosquitoes and other insect vectors under laboratory conditions.

## Figures and Tables

**Table 1:** The filariasis vectors identified during the study period.

Identified vectors	Species	No. of species	%
*Anopheles* mosquitoes	*An. gambiae* s.l.	221	21.84%
	*An. pharoensis*	207	20.45%
	*An. funestus*	193	19.07%

*Culex* mosquitoes	*Cx. antennatus*	190	18.77%
	*Cx. quinquefasciatus*	201	19.86%

Total		1012	100%

**Table 2:** Qualitative phytochemical screening of selected plant extract.

Phytochemical	*Allium sativum*			*Zingiber officinale*		
	Aqueous	Methanol	Ethanol	Aqueous	Methanol	Ethanol
Saponin	+	+	+	—	—	—
Phlobatannin	—	—	—	+	+	+
Tannin	+	+	+	+	+	+
Phenol	+	+	+	—	—	—
Anthraquinones	+	+	+	—	—	—
Flavonoid	+	+	+	+	+	—
Glycosides	+	+	+	+	+	+
Steroids	+	+	+	+	+	+
Terpenes	+	+	+	+	+	+

+ = present; — = absent.

**Table 3:** Larvicidal activity of crude extracts of *A. sativum* and *Z. officinale* against filariasis vectors.

Filariasis vectors	Con.	*Allium sativum*			*Zinger officinale*		
		Aqueous	Ethanol	Methanol	Aqueous	Ethanol	Methanol
*An. gambiae* s.l.	250 ppm	0 ± 0.00	1 ± 1.29	4 ± 3.12	0 ± 0.00	1 ± 1.29	4 ± 2.37
	500 ppm	0 ± 0.00	3 ± 3.02	6 ± 4.56	0 ± 0.00	3 ± 1.42	6 ± 2.49
	750 ppm	1 ± 1.29	4 ± 2.27	7 ± 2.45	1 ± 1.29	4 ± 1.22	7 ± 3.09

*An. pharoensis*	250 ppm	0 ± 0.00	2 ± 2.15	4 ± 1.13	0 ± 0.00	0 ± 0.00	1 ± 1.33
	500 ppm	1 ± 1.29	3 ± 2.89	6 ± 5.02	1 ± 1.29	2 ± 2.21	5 ± 3.76
	750 ppm	1 ± 1.29	6 ± 4.21	8 ± 2.33	1 ± 1.29	5 ± 2.16	7 ± 4.52

*An. funestus*	250 ppm	2 ± 1.11	1 ± 1.29	7 ± 4.14	1 ± 1.29	3 ± 1.07	6 ± 4.56
	500 ppm	4 ± 3.12	4 ± 3.12	9 ± 4.96	2 ± 2.15	6 ± 2.08	9 ± 4.17
	750 ppm	5 ± 3.45	7 ± 2.93	9 ± 4.67	2 ± 2.83	7 ± 1.2	9 ± 4.33

*Cx. antennatus*	250 ppm	0 ± 0.00	3 ± 1.21	6 ± 3.21	0 ± 0.00	1 ± 1.12	8 ± 3.97
	500 ppm	0 ± 0.00	5 ± 3.79	8 ± 2.74	1 ± 1.33	1 ± 1.33	8 ± 2.85
	750 ppm	2 ± 2.21	8 ± 4.12	9 ± 2.39	3 ± 2.75	4 ± 3.12	9 ± 2.75

*Cx. quinquefasciatus*	250 ppm	1 ± 1.91	2 ± 1.97	5 ± 1.29	3 ± 2.47	2 ± 2.21	5 ± 2.89
	500 ppm	3 ± 2.19	7 ± 2.19	6 ± 173	4 ± 2.11	6 ± 4.56	7 ± 3.91
	750 ppm	5 ± 3.22	9 ± 4.87	5 ± 1.32	4 ± 2.79	6 ± 3.35	8 ± 4.12

Mean ± standard deviation of three replicate. Con = concentration; ppm = part per million.

**Table 4:** Toxicity values of crude extracts of *A. sativum* and *Z. officinale* against filariasis vectors.

Filariasis vectors	Statistical analysis	*Allium sativum*			*Zinger officinale*		
		Aqueous	Ethanol	Methanol	Aqueous	Ethanol	Methanol
*An. gambiae* s.l.	*X* ^2^	7.0807	3.8095	3.8095	2.069	2.3864	1.9005
	*p* value	0.029	0.1489	0.1489	0.3554	0.3033	0.3867
	LC_50_	736 ppm	754 ppm	262 ppm	781 ppm	924 ppm	355 ppm

*An. pharoensis*	*X* ^2^	4.2857	3.7321	3.3333	1.0714	7.0807	0.9025
	*p* value	0.1173	0.1547	0.1889	0.5853	0.029	0.6368
	LC_50_	768 ppm	697 ppm	340 ppm	1940 ppm	728 ppm	393 ppm

*An. funestus*	*X* ^2^	2.0096	7.5	1.92	0.48	3.4821	3.75
	*p* value	0.3661	0.02352	0.3829	0.7866	0.1753	0.1534
	LC_50_	726 ppm	564 ppm	131 ppm	4259 ppm	415 ppm	195 ppm

*Cx. antennatus*	*X* ^2^	4.2857	5.0893	2.6087	4.0385	3.75	0.48
	*p* value	0.1173	0.0785	0.2713	0.1328	0.1534	0.7866
	LC_50_	768 ppm	422 ppm	196 ppm	919 ppm	1080 ppm	27 ppm

*Cx. quinquefasciatus*	*X* ^2^	3.8095	10.833	0.2678	0.2870	4.2857	2.1
	*p* value	0.1489	0.0044	0.8747	0.8663	0.1173	0.3499
	LC_50_	754 ppm	384 ppm	53 ppm	1679 ppm	501 ppm	251 ppm

ppm = parts per million; LC_50_ = lethal concentration; *X*^2^ = Chi-squared value; *p* value = probability value.

## Data Availability

All the data used to support the findings of this research were included in the manuscript.
